# Antimalarial Activity of 4-Metoxychalcones: Docking Studies as Falcipain/Plasmepsin Inhibitors, ADMET and Lipophilic Efficiency Analysis to Identify a Putative Oral Lead Candidate

**DOI:** 10.3390/molecules181215276

**Published:** 2013-12-10

**Authors:** Michael Eder de Oliveira, Gisele Cenzi, Renata Rachide Nunes, Carla Regina Andrighetti, Denia Mendes de Sousa Valadão, Cláudia dos Reis, Cláudia Maria Oliveira Simões, Ricardo José Nunes, Moacyr Comar Júnior, Alex Gutterres Taranto, Bruno Antonio Marinho Sanchez, Gustavo Henrique Ribeiro Viana, Fernando de Pilla Varotti

**Affiliations:** 1 Centro de Ciências da Saúde-UFSJ/Campus Centro-Oeste, CEP 35501-296, Divinópolis, MG, Brazil; 2 Instituto de Ciências da Saúde-UFMT/Campus Sinop, CEP 78557-267, Sinop, MT, Brazil; 3 Departmento de Ciências Farmacêuticas-UFSC/Campus Universitário Trindade, CEP 88040-900, Florianópolis, SC, Brazil; 4 Departmento de Química-UFSC/Campus Universitário Trindade, CEP 88040-900, Florianópolis, SC, Brazil

**Keywords:** 4-methoxychalcone, malaria, ADMET properties, LipE

## Abstract

Herein, we report the antimalarial activity of nine 4-methoxychalcone derivatives **1a**–**i** and an initial analysis of their ADMET properties. All compounds showed potent activity against the *P. falciparum* chloroquine-resistant clone W2, with IC_50_ values ranging from 1.96 µM to 10.99 µM, with moderate or low cytotoxicity against the HeLa cell line. The compound **1a** (IC_50_ = 2.06 µM) had the best selectivity index (9.0). All the sulfonamide 4-metychalcone derivatives synthesized had cLogP values between 2 and 5 (mean value 3.79) and molecular weights (MWs) below 500. The substitution of the pyrrolidine group in **1i** by a morpholine group in **1a** reduced the cLogP value from 3.05 in compound **1i** to 2.34 in compound **1a.** Indeed, compound **1a** had the highest LipE value. The binding free energy of compound **1a** showed it to be the most optimal chalcone derivative for plasmepsin-2 (−7.3 Kcal/mol). The physicochemical properties and LipE analysis of the dataset allowed us to establish that compound **1a** is the highest quality compound of the series and a potential oral lead candidate.

## 1. Introduction

In 2010, there were an estimated 219 million cases of malaria, a global infectious disease, and it was responsible for 660,000 deaths [1]. The arsenal of antimalarial drugs is limited and currently the most effective treatment against the pathogenic agent *Plasmodium falciparum* includes artemisinin combination therapies (ACTs), although resistance to artemisinins has been reported in four countries of the South-East Asia region: Cambodia, Myanmar, Thailand and Vietnam [2,3].

The need for new antimalarial therapies has stimulated research into the search for synthetic molecules that are effective against acquired resistance to artemisinin derivatives and would result in promising antimalarial activity [4,5]. In this context, chalcones (1,3-diaryl-2-propen-1-ones), the bio-precursors of flavonoids, have been used as scaffolds for medicinal chemists for many years and chalcone analogs are associated with a broad range of biological activities, including antimalarial activity [6,7,8,9].

The antimalarial activity of chalcones is associated with inhibiting either plasmodial aspartate proteases or cysteine proteases [10]. Plasmodial aspartate and cysteine proteases are attractive targets for antimalarial therapy due to their role in the degradation of hemoglobin during erythrocytic parasite development [11]. In addition, the sulfonamide group and its derivatives have already shown antimalarial activity [12]. A recent strategy adopted in the search for new antimalarial drugs is the design of hybrids [13,14].

One of the main challenges in the development of new antimalarial drugs is how to achieve a viable lead candidate with good pharmacokinetic properties: absorption, distribution, metabolism, excretion and toxicity (ADMET) [15].

**Figure 1:**
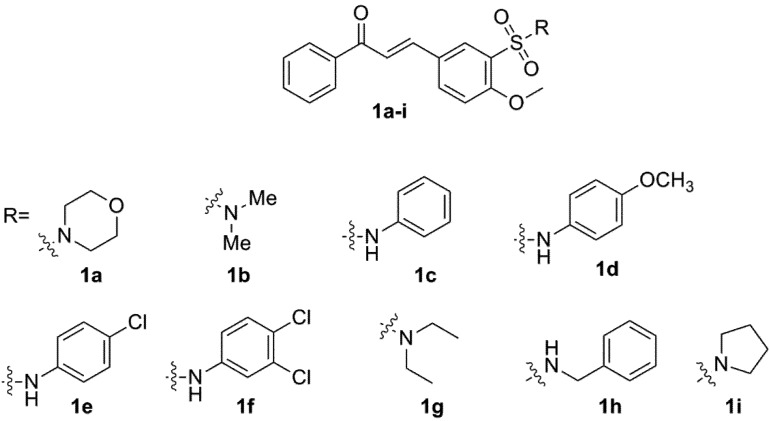
4-Methoxychalcone derivatives **1a**–**i** synthesized [17].

It is well known that artemisinin derivatives act only for a short time and require frequent dosing to maintain efficacy [16]. Therefore, the search for artemisinin derivatives, as well new synthetic compounds with enhanced pharmaceutical properties, has received considerable attention [5]. Herein we report the antimalarial activity of 4-methoxychalcone derivatives **1a**–**i** (Figure 1) [17], molecular docking studies and an initial analyses of their ADMET properties. This study led to the selection of a lead candidate with optimal oral absorption properties.

## 2. Results and Discussion

### 2.1. Biological Activity

The half maximal inhibitory concentration (IC_50_) and the lethal drug concentration (LC_50_) values determined for nine 4-metoxychalcone derivatives **1a**–**i**, are given in Table 1. All compounds showed potency against the *P. falciparum* chloroquine-resistant clone W2, with IC_50_ values ranging from 1.96 µM to 10.99 µM. In parallel we tested the toxicity of the compounds against the HeLa cell line. All LC_50_ values were higher than the IC_50_ values. The compound **1a** had the best selectivity index (9.0) and was advanced for more detailed physicochemical analysis.

**Table 1:** *In vitro* antiplasmodial activity (IC_50_), cytotoxicity (LC_50_) and selectivity index (SI) of the 4-methoxychalconederivatives**1a**–**i**.

Compound	IC_50_ (mM)	LC_50_ (mM)	SI
1a	2.06 ± 0.05	18.58 ± 1.26	9.0
1b	5.79 ± 0.12	20.70 ± 1.62	3.6
1c	2.03 ± 0.28	11.54 ± 0.23	5.7
1d	3.64 ± 0.40	11.07 ± 1.96	3.0
1e	2.83 ± 0.21	10.49 ± 2.27	3.7
1f	4.33 ± 0.06	18.97 ± 0.43	4.4
1g	10.99 ± 0.57	20.70 ± 1.60	1.9
1h	1.96 ± 0.02	9.42 ± 2.09	4.8
1i	2.07 ± 0.22	9.18 ± 1.51	4.4
CQ ^a^	0.45 ± 0.04	>100	>100

^a ^chloroquine

### 2.2. Physicochemical Properties Analysis

A recent antimalarial screening with 88 synthesized chalcones showed that the majority of the compounds were active *in vitro* against *P. falciparum* with low cytotoxicity. However, they lacked oral bioavailability due to poor ADMET properties [10]. Compounds that have become marketed oral drugs have physicochemical characteristics that favor drug absorption or permeability [18].

These findings prompted us to evaluate the physicochemical properties of our chalcone derivatives (Table 2) to identify compounds with optimal oral absorption properties and to guide future structural modifications in order to improve ADMET properties.

**Table 2:** Physicochemical properties of 4-methoxychalcone derivatives **1a**–**i**.

Compound	Molecular Weight	pIC_50_	cLog P	LipE ^a^
1a	387.45	5.685	2.34	3.345
1b	345.41	5.237	2.74	2.497
1c	393.46	5.692	3.87	1.822
1d	423.48	5.439	3.74	1.699
1e	427.90	5.549	4.42	1.129
1f	462.35	5.364	4.98	0.384
1g	387.49	4.959	4.83	0.129
1h	407.48	5.707	4.17	1.537
1i	371.45	5.683	3.05	2.633
CQ ^b^	515.86	6.343	3.73	2.613

^a ^LipE = pIC50 – cLogP; ^b ^chloroquine

There is a general consensus that the molecular properties of drug candidates, such as molecular weight and lipophilicity, are important ADMET properties [19]. Other properties such as solubility, clearance and volume of distribution are also important to consider in the profile of drug candidates [20]. In addition, the use of computational tools has contributed to improve drug design in order to attain more efficacious antimalarial activity [21,22].

To rationalize the drug discovery/development process and to guide the optimization from lead compound to successful drug candidate, rules for predicting dug-like physio-chemical properties have been introduced [23]. The Lipinski’s ‘Rule of Five’ [24] as well as other parameters like lipophilic efficiency (LipE) [25] have been shown to be useful tools to aid in choosing oral drug candidates.

**Figure 2:**
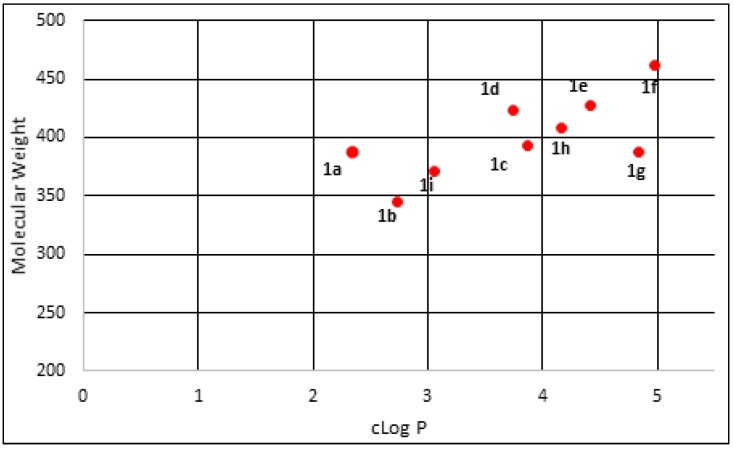
cLogP *versus* molecular weight distribution of synthesized 4-methoxychalcone derivatives.

All the sulfonamide 4-methylchalcone derivatives synthesized had cLogP values between 2 and 5 (mean value 3.79) and molecular weights (MWs) below 500 (mean value 400.72). Gleeson *et al.* [26] suggested that compounds with a cLogP < 4 and MWs below 400 Da have a more favorable ADMET profile. Figure 2 shows the distribution of cLogP *versus* MW for the nine synthesized compounds. Among them, five compounds (~56%) had a cLogP between 2 and 4.

In addition to a range of lipophilicity and MW it is also desirable that drug candidates have high *in vitro* potency. High potency compounds reduce the chances that a drug will lack specificity for its target and allows the administration of smaller doses, thus reducing the risk of adverse effects [19].

Although *in vitro* potency and lipophilicity of compounds are important parameters to evaluate, the concept of Lipophilic Efficiency (LipE) aids in establishing a more balanced relationship between the potency observed *in vitro* and lipophilicity properties of evaluated chemical compounds [27]. Ryckmans *et al.* [19] reported that high quality lead compounds possess higher LipE values.

**Figure 3:**
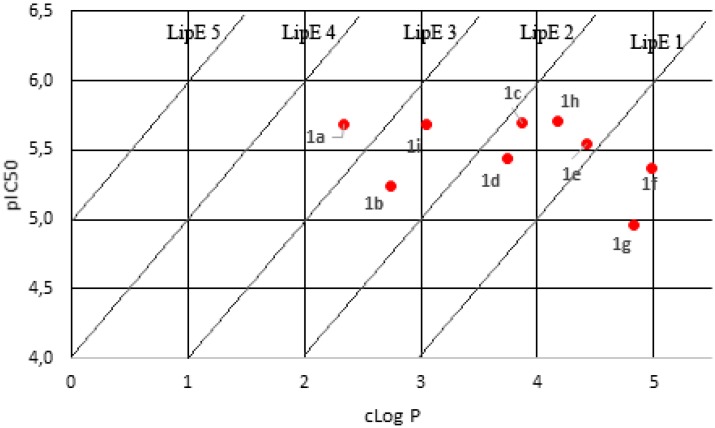
cLogP *versus* pIC_50_ plot and LipE analysis.

Plotting cLogP against pIC_50_ (Figure 3) for the nine sulfonamide 4-methylchalcone derivatives showed a distribution along lines of identical LipE values. Compounds **1c**–**h** have low LipE values (higher cLogP values).

**Figure 4:**
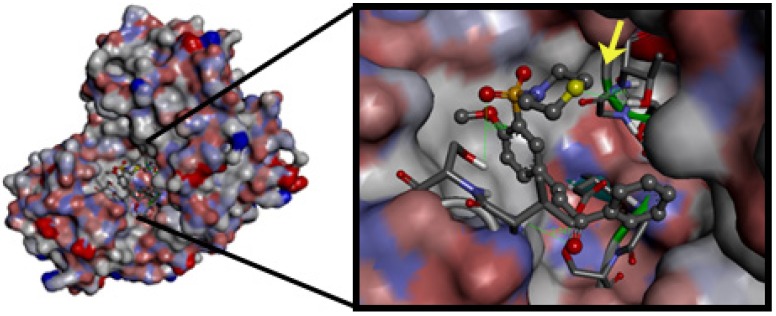
Compound **1a** docked into the plasmepsin-2 (PL-2) binding site, generated by AutoDock Vina and viewed by Discovery Studio Client (version 3.1.1.11157). Highlighting the H-bond with residue Thr217 and oxygen atom of the morpholin ring at the binding site (indicated by arrow).

**Figure 5:**
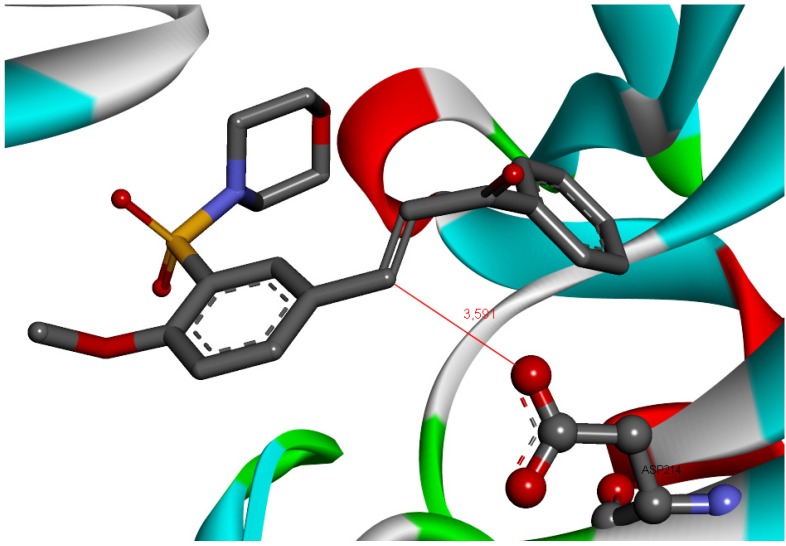
Compound **1a** docked into the plasmepsin-2 (PL-2) binding site. Highlighting the the α,β-unsaturated carbonyl moiety (Michael acceptor) and carboxylate anion (Michael donor) of Asp214.

It is interesting to observe that substitution of the pyrrolidine group in **1i** by a morpholine group in **1a** had similar potency, but the cLogP value of compound **1i** was reduced from 3.05 to 2.34 in compound **1a**. Recent animal safety studies demonstrated that the risk of side effects/toxicity is reduced for compounds with cLogP below 3 [28]. In addition, this finding suggests that the presence of polar groups such as the oxygen atom of the morpholine ring may be important for the binding site interactions of compound **1a**. Indeed compound **1a** had the highest LipE value of the data set and was deemed to be the most optimal compound. The docking results also showed an additional binding interaction of compound **1a** with the plasmepsins due the presence of a morpholine ring. This additional binding interaction of compound **1a** with plasmepsin-2 is shown in Figure 4. In addition, docking results suggests a Michael reaction mechanism between these ligands and proteases. Figure 5 highlights a short distance (3.59 Å) between the α,β-unsaturated carbonyl moiety (Michael acceptor) of **1a** and carboxylate anion (Michael donor) of Asp214, suitable for a nucleophilic attack resulting in an alkylation process [29].

### 2.3. Docking of 4-Metoxychalcone Derivatives to Falcipain-2, Plasmepsin-2, and Plasmepsin-4

Chalcones have been reported to inhibit plasmodial aspartate proteases and cysteine proteases [10]. We therefore performed docking studies to determine the binding orientations of the 4-methoxychalcones at the active sites of amino acid residues of the enzymes falcipain-2, plasmepsin-2 and plasmepsin-4. The binding free energy of the docked compounds is given in Table 3.

**Figure 6:**
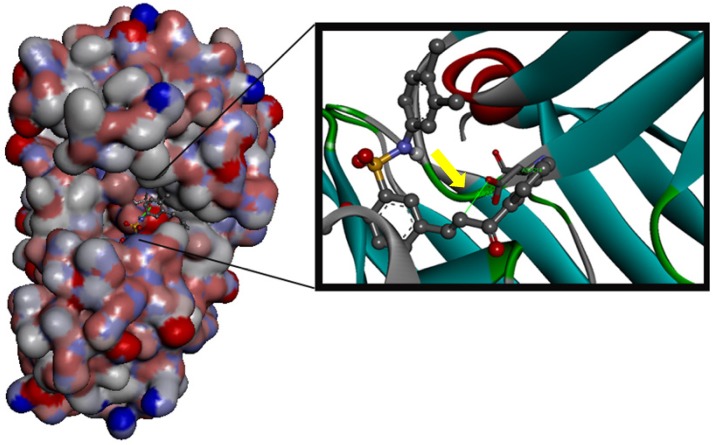
Compound **1f** docked into the plasmepsin-2 (PL-2) binding site, generated by AutoDock Vina and viewed by Discovery Studio Client (version 3.1.1.11157). The α,β-unsaturated ketone system of chalcones acts as a Michael acceptor with residue Asp241 at the binding site (indicated by arrow).

The binding free energy of compound **1f**, determined to be the most optimal chalcone derivative, for falcipain-2 (−6.5 Kcal/mol), plasmepsin-4 (−7.6 Kcal/mol) and plasmepsin-2 (−8.5 Kcal/mol) showed a better interaction for aspartic proteases (Figure 6), although, compound **1f** does not exhibit the best physical chemistry parameters for oral administration (Table 3).

**Table 3:** Molecular docking of 4-metoxychalcone derivatives **1a**–**i** to falcipain-2, plasmepsin-2 and plasmepsin-4.

Compound	ΔG Energy (Kcal.mol^−1^)		
	Falcipain-2	Plasmepsin-2	Plasmepsin-4
**1a**	−4.9	−7.3	−6.9
**1b**	−5.5	−7.2	−7.0
**1c**	−6.2	−8.0	−7.4
**1d**	−6.1	−7.8	−7.3
**1e**	−6.1	−8.0	−7.5
**1f**	−6.5	−8.5	−7.6
**1g**	−5.5	−6.6	−6.2
**1h**	−5.0	−7.7	−6.9
**1i**	−5.5	−7.1	−7.1

## 3. Experimental

### 3.1. Drug Samples

The compounds were stored at −20 °C as 10 mg/mL stock solutions in dimethylsulfoxide (DMSO) (Sigma, St. Louis, MO, USA). The compounds were diluted in DMSO and used at a final concentration of 0.01% (v/v).

### 3.2. Biological Assays

#### 3.2.1. *Plasmodium Falciparum* Continuous Culture

Human red blood cells infected with the *P. falciparum* clone W2 (chloroquine-resistant) were maintained in continuous culture as previously described [30,31]. The human red blood cells and plasma were ceded by the Fundação Hemominas-Hemonúcleo Divinópolis [32]. The synchronization of the parasites was achieved by sorbitol treatment [33] and parasitaemia was determined microscopically in Giemsa-stained smears.

#### 3.2.2. *In vitro* Antiplasmodial Activity

The antimalarial effect of the synthetic 4-methoxychalone sulfonamide derivatives and the control compound chloroquine was measured using traditional methods [34] with minor modifications. Briefly, ring-stage parasites in sorbitol-synchronized blood cultures were added to 96-well culture plates with 2% parasitaemia and 2% haematocrit and then incubated with the test compounds. The compounds were diluted in complete media, from 10 mg/mL stock solutions in DMSO, at various concentrations (0.1–30 µM). After 48 h incubation, Giemsa-stained blood smears were taken for microscopic evaluation of parasitaemia. All experiments were performed in triplicate. The results are expressed as the mean of the IC_50_ (the lethal drug concentration that reduced parasite viability by 50%).

#### 3.2.3. Cytotoxicity Assay

The cytotoxicity of the compounds was assessed with the HeLa human cell line (cervix adenocarcinoma ATCC# CCl-2) using the MTT (3-(4,5-dimethylthiazol-2-yl)-2,5-diphenyltetrazolium bromide) (Sigma, St. Louis, MO, USA) colorimetric method. Briefly, the cells were plated in 96-well plates (1 × 10^5^ cells/well) and incubated for 24 h at 37 °C in a humidified atmosphere with 5% CO_2_. After 24 h, the wells were washed with culture medium (RPMI + 10% inactivated fetal calf serum + 2 mM L-glutamine) and incubated with the compounds at various concentrations (0.05 to 500 µM). After 48 h incubation, the plates were treated with MTT. The colorimetric reading was performed in a microplate reader Spectramax M5e (Molecular Devices, Sunnyvale, CA, USA) at 550 nm. Cytotoxicity was scored as the percentage reduction in absorbance *versus* untreated control cultures [35,36]. All experiments were performed in triplicate. The results are expressed as the mean of the LC50 (the lethal drug concentration that reduced cell viability by 50%). The IC_50_ and LC50 values were calculated using OriginPro8.0 (OriginLab Corporation, Northampton, MA, USA) software. A selectivity index (SI), corresponding to the ratio between the cytotoxic and antiparasitic activities of each compound, was calculated as follows:

SI = LC_50_ HeLa/IC_50 _*Plasmodium falciparum*

### 3.3. General Procedure of Docking

Computational flexible docking and redocking was executed with the AutoDock Vina software. The docking procedure involved the preparation of the ligand and macromolecules using simulation boxes sufficiently large to involve the entire region of interaction between the ligand and receptor [37]. All compounds were docked to catalytic binding sites of falcipain-2 **(**PDB 3BPF), plasmepsin-2 (PDB 1LF3) and plasmepsin-4 (PDB 2ANL) to predict their binding modes and approximate binding free energies. The structures of all ligands were minimized using Gaussian09 software with PM6 method [38]. The lowest energy conformation was identified and binding energies were evaluated. cLogP values of the compounds was estimated using ChemBioDraw Ultra software version 12.0.

### 3.4. Statistical Analysis

The average IC_50_s and LC_50_s were compared using ANOVA. Difference between the values was evaluated using GraphPad Prism 5 Demo. Statistical significance was defined at the 5% level (*p* < 0.05).

## 4. Conclusions

All assayed compounds showed antimalarial activity. Among the nine compounds assayed there were four in the same potency range. The physicochemical and LipE analysis of the dataset allowed us to establish that compound **1a** is the highest quality compound of the series. Molecular docking provided insight into the binding modes of compound **1a** into the PL2 binding site. The data indicated that the presence of a morpholine ring as a substituent is required for optimal binding site interaction. Further optimization of this lead compound may provide a more potent and selective plasmepsin inhibitor potentially with better druggabilty.
